# Timing the clot: circadian rhythms in hemostasis

**DOI:** 10.1016/j.rpth.2026.106848

**Published:** 2026-07-14

**Authors:** Gael B. Morrow

**Affiliations:** Centre for Biomedical & Biotechnology Research, School of Pharmacy, Applied Sciences & Public Health, Robert Gordon University, Aberdeen, United Kingdom

The circadian rhythm is the body’s natural internal biological clock, or master clock. It is a continuous, endogenous 24-hour oscillation that synchronizes essential physiological processes with the external light-dark cycle [1]. The hypothalamic suprachiasmatic nucleus is the central circadian pacemaker [2]. The suprachiasmatic nucleus coordinates a network of oscillators (peripheral clocks) across the body’s tissues and organs [2,3]. At the molecular level, the circadian system is regulated by 2 key transcription factors: brain and muscle ARNT-like 1 and circadian locomotor output cycles protein kaput [4,5]. Disruption of the circadian rhythm can result from behavioral misalignment, sleep disorders, genetic alterations, and lifestyle or environmental factors [6,7].

The relationship between the circadian rhythm and hemostasis has been extensively researched over the past decades. A breakthrough in the field occurred in 1963, when Pell and D’Alonzo [8] described a pronounced circadian pattern in the onset of acute myocardial infarction, finding that it was most likely to occur in the morning hours. Later studies investigated platelet activity, coagulation and fibrinolysis factor levels, and changes in blood pressure, indicating a prothrombotic state occurs during the early hours of the morning [9,10].

In the latest issue of Research and Practice in Thrombosis and Haemostasis, Ibrahim et al. [11] conduct a systematic review of the regulation of hemostasis by the circadian clock. In this analysis, they accessed 26 studies separated into 4 themes: 1) evidence of circadian rhythms in hemostasis and platelet function, 2) genetic and pharmacologic modulation of circadian clock genes and their impact on thrombosis, 3) circadian gene expression in platelets and megakaryocytes, and 4) external and lifestyle factors influencing circadian regulation of hemostasis (Figure).

**Figure:**
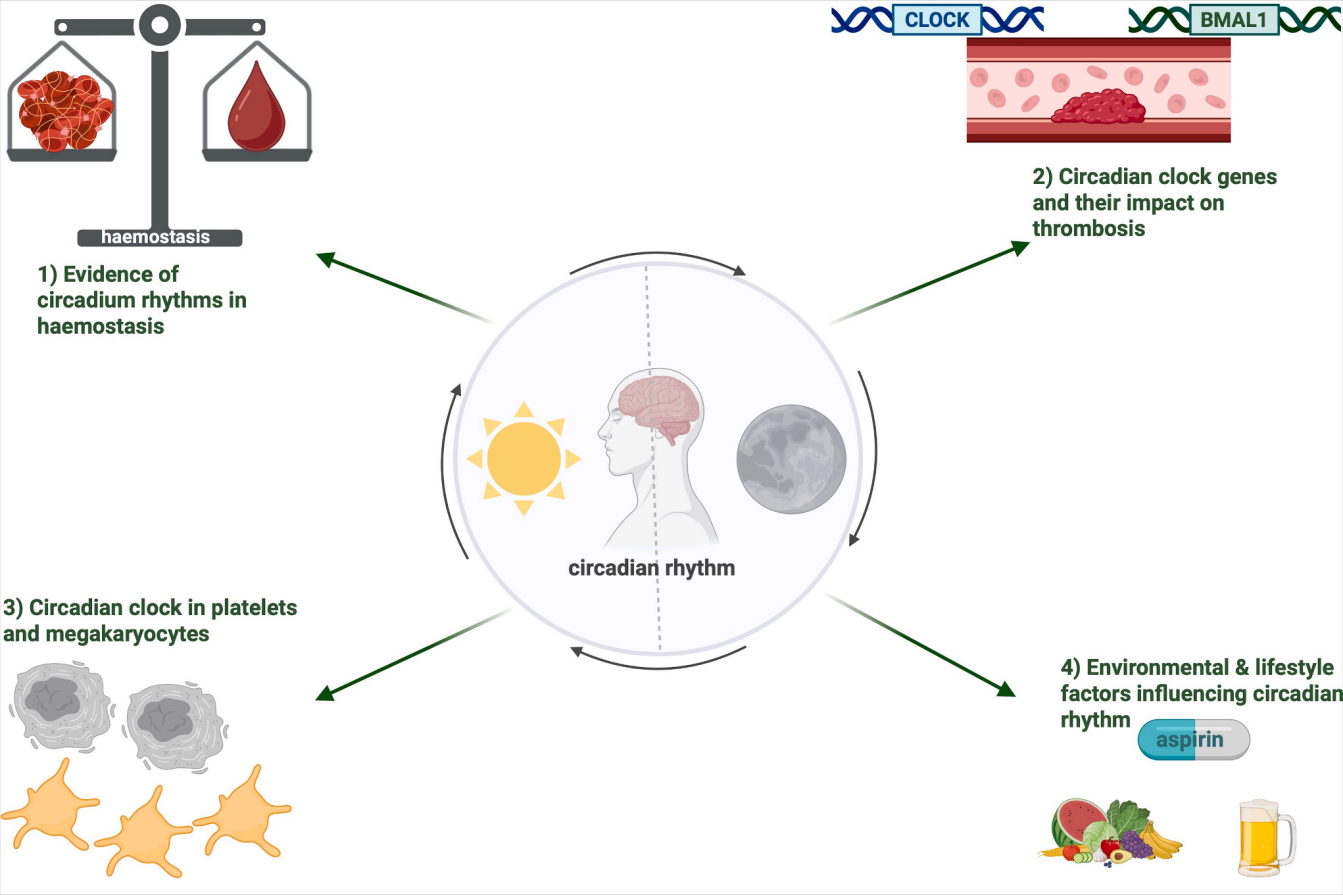
Factors influencing circadian rhythm in hemostasis. BMAL1, brain and muscle ARNT-like 1; CLOCK, circadian locomotor output cycles protein kaput.

Across the studies included in the review, 91.6% reported findings that circadian rhythm influences hemostasis, with only 1 study reporting no circadian variation. The studies used diverse populations and experimental models: human blood samples collected at specific time points throughout the day; investigations of circadian effects in shift workers (night vs day); and animal studies that simulated light-dark cycles. The variability across studies highlights the need for standardized circadian protocols in future hemostasis research.

Studies evaluating circadian clock genes have found consistent evidence of the role of circadian clock components in regulating hemostasis, thrombosis, and platelet function. Only 2 studies investigated platelets and megakaryocytes; however, the results were novel and revealed compelling evidence that megakaryocytes possess their own circadian clock. Environmental and lifestyle factors, including the timing of aspirin administration, alcohol consumption, and following a keto diet, were found to entrain or disrupt hemostatic regulation through a variety of mechanisms, including clock-gene modulation and platelet signaling pathways.

This review [11] has highlighted substantial progress and exciting contributions to the field of hemostasis and thrombosis in elucidating the molecular mechanisms of the circadian clock and its influence on hemostasis. However, important questions remain: how does regulation of the circadian clock translate into clinically meaningful changes in thrombotic risk? Future studies will continue to elucidate the molecular and cellular mechanisms of the circadian clock and its clinical role in hemostasis.
